# Circulating branch chain amino acids and improvement in liver fat content in response to exercise interventions in NAFLD

**DOI:** 10.1038/s41598-021-92918-1

**Published:** 2021-06-28

**Authors:** Xiulin Shi, Hongyan Yin, Jia Li, Caoxin Huang, Yinling Chen, Zheng Chen, Wei Liu, Weijuan Su, Yiping Zhang, Mingzhu Lin, Yan Zhao, Xuejun Li

**Affiliations:** 1 Department of Endocrinology and Diabetes, Xiamen Diabetes Institute, The First Affiliated Hospital of Xiamen University, No.55 Zhenhai Road, Xiamen, 361003 China; 2 Xiamen Clinical Medical Center for Endocrine and Metabolic Diseases, Xiamen, China; 3 Fujian Province Key Laboratory of Diabetes Translational Medicine, Xiamen, China; 4 Xiamen Diabetes Prevention and Treatment Center, Xiamen, China

**Keywords:** Endocrine system and metabolic diseases, Obesity

## Abstract

Nonalcoholic fatty liver disease is likely to be associated with increased circulating branched-chain amino acids. We investigated the relationship between changes in branched-chain amino acids levels in the serum and improvement in liver fat content caused by exercise intervention in individuals with nonalcoholic fatty liver disease. The exploratory study included 208 central obesity and nonalcoholic fatty liver disease individuals from an exercise intervention randomized clinical trial for nonalcoholic fatty liver disease. The participants were randomly assigned to control, moderate, and vigorous-moderate exercise groups for 12 months. Changes in total branched-chain amino acids, leucine, isoleucine, and valine levels from baseline to 6 months were calculated. Liver fat content was determined by proton magnetic resonance spectroscopy. Reductions in circulating levels of total branched-chain amino acids, leucine, and valine levels from baseline to 6 months were significantly associated with the improvement of liver fat content at 6 months and 12 months (*p* < 0.01 for all) after adjustments for age, sex, total energy intake, protein intake, intervention groups, HOMA-IR, BMI, liver fat content, total branched-chain amino acids, leucine, and valine at baseline, respectively. These associations were still significant after further adjustments for changes in HOMA-IR and BMI from baseline to 6 months (*p* < 0.05 for all). Our findings indicated that reductions in circulating branched-chain amino acids levels were associated with an improvement in liver fat content by exercise intervention among patients with nonalcoholic fatty liver disease, which was independent of changes in BMI or HOMA-IR.

## Introduction

Nonalcoholic fatty liver disease (NAFLD) has rapidly become a global pandemic disease^1^, which is related to obesity, type 2 diabetes, and cardiovascular disease^2–4^. Accumulating evidence has demonstrated the increased circulating levels of branched-chain amino acids (total BCAAs, leucine, isoleucine, and valine) in NAFLD and nonalcoholic steatohepatitis (NASH)^5–7^. Significant changes in hepatic BCAAs composition and transcriptomic metabolism profiles during the progression of NAFLD have also been reported^8^. Steatosis around the central vein (zone 3) hepatocytes is one of the pathological features of NAFLD/NASH. The levels of BCAAs have been shown to be associated with the lipid droplets heterogeneity of hepatocytes around zone 3 in patients with NAFLD/NASH. Moreover, a diet with high level of BCAAs could induce heterogeneity in the lipid droplets of NAFLD model mice, which indicated that impaired BCAAs catabolism might contribute to the pathogenesis of NAFLD/NASH, and amelioration of the impaired catabolism might function as a new therapeutic approach for NAFLD^9,10^.

Numerous studies have demonstrated that exercise is a safe and economic choice to treat or prevent NAFLD^11–13^. However, the underlying mechanism of beneficial effects of exercise on NAFLD remains not fully elucidated. Skeletal muscle, the initial site for BCAAs catabolism, could also release alanine and glutamine into circulation. Physical exercise is associated with enhanced BCAAs oxidation and glutamine release from muscles^14^. Ananimal study suggested that branched-chain-amino-acid aminotransferase (BCKD), the first enzyme in the BCAAs catabolism, is activated within the muscles during exercise^15^. In healthy adult males, plasma BCAAs concentrations were decreased by an acute exercise bout in both athletes and untrained subjects^16^. In light of the association between BCAAs and exercise, it may be rational to predict a potential correlation between the amelioration effects of exercise on liver steatosis and the change of BCAAs levels in NAFLD. However, to the best of our knowledge, there is still a lack of comprehensive study analyzing the association between change in BCAAs levels and improvement in liver fat content caused by exercise interventions in NAFLD population.

Therefore, in the present study, we determined the changes in BCAAs levels and further analyzed its association with improvement in liver fat content among NAFLD from the effects of Moderate and Vigorous Exercise on Nonalcoholic Fatty Liver Disease, a1-year randomized trial^11^.

## Results

Among the 208 participants included in the analysis, the mean age was 53.9 years, 149 (67.7%) participants were female. Table 1 showed the baseline characteristics of participants in the study according to the terciles of changes in total BCAAs levels from baseline to 6 months. For total BCAAs levels, the median (25th, 75th) μg/mL for each tertile was tertile 1: − 5.6 (− 7.9, − 2.8) μg/mL; tertile 2:1.7 (0.4, 2.8) μg/mL; and tertile 3:6.4 (4.8, 8.6) μg/mL, respectively. Participants in the lowest tertile group (the largest reduction) of total BCAAs had a higher BMI (28.8vs 27.4 kg/m^2^, *p* < 0.001), total BCAAs (50.83 vs. 42.3 μg/mL, *p* < 0.001), isoleucine (8.1 vs. 6.6 μg/mL, *p* < 0.001), leucine (17.0 vs. 14.3 μg/mL, *p* < 0.001) and valine (26.6 vs. 21.3 μg/mL, *p* < 0.001) compared to those in highest tertile group (the less reduction). No significant differences were observed for age, sex, education, cigarette smoking, alcohol drinking, blood pressure, weight, glucose, or liver fat content among the tertiles of change in total BCAAs levels. At the baseline before the exercise intervention, levels of total BCAAs, isoleucine, leucine, and valine levels were not significantly associated with liver fat content in three models (Table 2), visceral fat, and subcutaneous fat (Table 3).

**Table 1:** Baseline characteristics according to the tertiles of change in serum total BCAAs^a^ levels.

	Change in total BCAAs levels, μg/mL			*P *_*trend*_
	Tertile 1(< − 1.2)	Tertile 2 (− 1.2–3.9)	Tertile 3 (≧3.9)	
Participants, n	69	70	69	
Age, years	54.0 (7.8)	53.8 (6.6)	53.8 (6.7)	0.990
Female, n (%)	41 (59.4)	52 (74.3)	48 (69.6)	0.160
Intervention groups				< 0.001
Control, n (%)	24 (34.8)	16 (22.7)	32 (46.3)	
Moderate exercise, n (%)	32 (46.4)	28 (40.0)	26 (37.1)	
Vigorous-moderate exercise, n (%)	13 (18.8)	26 (37.1)	28 (40.5)	
High school education, no (%)	20 (29.0)	18 (25.7)	19 (27.5)	0.910
Current cigarette smoking, no. (%)	17 (26.4)	12 (17.1)	13 (18.8)	0.514
Current alcohol drinking, no. (%)	23 (33.3)	16 (22.8)	24 (34.8)	0.247
Total energy intake, kcal/d^a^	2217.8 (480.6)	2083.0 (436.0)	2086.0 (438.5)	0.137
Fat intake, %	33.5 (7.6)	32.6 (6.9)	31.8 (6.1)	0.356
Protein intake, %	14.3 (3.0)	14.9 (4.0)	14.9 (3.0)	0.338
Body mass index, kg/m^2^	28.8 (3.6) **	27.7 (2.7)	27.4 (2.4)	0.013
Blood pressure, mmHg				
Systolic	133.7 (14.7)	133.1 (15.2)	131.5 (16.2)	0.678
Diastolic	81.8 (10.0)	80.4 (9.1)	79.5 (10.3)	0.388
Plasma glucose, mg/dL	104.7 (10.0)	103.7 (9.7)	102.6 (9.9)	0.468
HOMA-IR	3.3 (2.5–4.6)	3.4 (2.5–4.4)	3.2 (2.4–4.5)	0.386
Total BCAAs, μg/mL	50.8 (9.1) **	43.1 (6.5)	42.3 (7.6)	< 0.001
Isoleucine, μg/mL	8.1 (1.9) **	6.7 (1.3)	6.6 (1.4)	< 0.001
Leucine, μg/mL	17.1 (3.3) **	14.6 (2.2)	14.3 (2.6)	< 0.001
Valine, μg/mL	26.6 (4.3) **	21.7 (3.2)	21.3 (3.9)	< 0.001
Visceral fat	139.9 (43.3)	135.0 (36.0)	135.7 (49.3)	0.776
Subcutaneous fat	244.2 (88.5)	233.6 (70.7)	227.6 (63.3)	0.418
Liver fat content, %	17.2 (10.3–25.6)	14.5 (10.4–23.0)	13.7 (9.9–25.6)	0.165

Data are presented as mean (SD), unless otherwise indicated. a: Tertiles were categorized based on change in total BCAAs level from baseline to 6 months. Abbreviations: *IQR* interquartile range; *HOMA-IR* homeostatic model assessment of insulin resistance. **: *p* < 0.01 vs. Tertile3.

**Table 2:** Associations of liver fat content and total BCAAs, Isoleucine, Leucine, or Valine levels before exercise intervention among individuals with nonalcoholic fatty liver disease.

	Baseline total BCAAs		Baseline Isoleucine		Baseline Leucine		Baseline Valine	
	ß (SE)	*p*	ß (SE)	*p*	ß (SE)	*p*	ß (SE)	*p*
Model 1	0.11 (0.09)	0.223	0.82 (0.51)	0.109	0.32 (0.27)	0.240	0.19 (0.19)	0.306
Model 2	0.05 (0.10)	0.590	0.48 (0.52)	0.362	0.20 (0.27)	0.471	0.05 (0.19)	0.813
Model 3	0.03 (0.10)	0.791	0.37 (0.52)	0.478	0.12 (0.27)	0.658	0.01 (0.19)	0.965

ß (SE) represents changes of the outcomes per change in the circulating metabolite levels during the exercise intervention. Model 1: adjusted for age, sex, total energy intake, protein intake, and intervention groups; Model 2: adjusted for covariates in Model 1 + HOMA-IR; Model 3: adjusted for covariates in Model 2 + BMI. Abbreviations: *HOMA-IR* homeostatic model assessment of insulin resistance; *BMI* body mass index.

**Table 3:** Associations of adiposity measures and total BCAAs, Isoleucine, Leucine, or Valine before exercise intervention among individuals with nonalcoholic fatty liver disease.

	Baseline total BCAAs		Baseline Isoleucine		Baseline Leucine		Baseline Valine	
	ß (SE)	*p*	ß (SE)	*p*	ß (SE)	*p*	ß (SE)	*p*
Visceral fat	0.36 (0.36)	0.314	1.11 (1.96)	0.571	0.84 (1.04)	0.421	0.87 (0.71)	0.217
Subcutaneous fat	0.79 (0.63)	0.215	1.53 (3.46)	0.658	1.92 (1.84)	0.298	1.97 (1.25)	0.114

ß (SE) per unit of change in total BCAAs, Isoleucine, Leucine, or Valine for differences in the adiposity measures after adjustment for age, sex, total energy intake, protein intake, intervention group, HOMA-IR, and BMI. Abbreviations: *HOMA-IR* homeostatic model assessment of insulin resistance; *BMI* body mass index.

At 6 months, the median (25th,75th) μg/mL of changes in total BCAAs, isoleucine, leucine, and valine levels among total participants were 1.71 (− 2.8 to 4.74, *p* = 0.030) μg/mL, 0.16 (− 0.55 to 0.87, *p* = 0.082) μg/mL, 0.5 (− 0.73 to 1.86, *p* = 0.002)μg/mL and 0.39 (− 1.77 to 2.41, *p* = 0.129) μg/mL, respectively. We found that reductions in total BCAAs, isoleucine, leucine, and valine levels were significantly associated withtheloss in liver fat contentat 6 months after adjustment for age, sex, total energy intake, protein intake, intervention group, HOMA-IR, BMI, liver fat content at baseline, and total BCAAs, isoleucine, and valine levels at baseline (Model 1, ß(SE) for total BCAAs: 0.26(0.08), *p* < 0.001; ß(SE) for isoleucine: 0.98(0.40), *p* = 0.014; ß(SE) for leucine: 0.66(0.21), *p* = 0.005; ß(SE) for valine: 0.53(0.15), *p* < 0.001). Those associations were also significant after further adjustment for change in HOMA-IR from baseline to 6 months (Model 2, ß(SE) for total BCAAs: 0.21(0.07), *p* = 0.004; ß(SE) for leucine: 0.53(0.23), *p* = 0.022; ß(SE) for valine: 0.46(0.14), *pp* < 0.001), and change in BMI from baseline to 6 months (Model 3, ß(SE) for total BCAAs: 0.19(0.07), *p* = 0.012; ß(SE) for leucine: 0.48(0.23), *p* = 0.036; ß(SE) for valine: 0.40(0.14), *p* = 0.003) (Table 4).

**Table 4:** Changes (Δ) in liver fat content at 6 months and 12 months per unit of change in BCAAs levels at 6 months.

	Δtotal BCAAs		ΔIsoleucine		ΔLeucine		ΔValine	
	ß (SE)	*p*	ß (SE)	*p*	ß (SE)	*p*	ß (SE)	*p*
**At 6 months**								
Model 1	0.26 (0.08)	< 0.001	0.98 (0.40)	0.014	0.66 (0.21)	0.005	0.53 (0.15)	< 0.001
Model 2	0.21 (0.07)	0.004	0.66 (0.40)	0.097	0.53 (0.23)	0.022	0.46 (0.14)	< 0.001
Model 3	0.19 (0.07)	0.012	0.50 (0.40)	0.207	0.48 (0.23)	0.036	0.40 (0.14)	0.003
**At 12 months**								
Model 1	0.30 (0.08)	< 0.001	1.20 (0.40)	0.003	0.90 (0.23)	< 0.001	0.54 (0.14)	< 0.001
Model 2	0.28 (0.08)	< 0.001	1.11 (0.41)	0.007	0.85 (0.24)	< 0.001	0.51 (0.15)	< 0.001
Model 3	0.25 (0.08)	0.001	0.93 (0.41)	0.024	0.80 (0.23)	< 0.001	0.45 (0.01)	0.001

Model 1: adjusted for age, sex, total energy intake, protein intake, intervention groups, HOMA-IR, BMI, liver fat content at baseline, total BCAAs, isoleucine, leucine, and valine at baseline respectively; Model 2: adjusted for covariates in Model 1 + ΔHOMA-IR; Model 3: adjusted for covariates in Model 2 + ΔBMI. Abbreviations: *HOMA-IR* homeostatic model assessment of insulin resistance; *BMI* body mass index. ΔHOMA-IR and ΔBMI: changes in HOMA-IR and BMI from baseline to 6 months.

We further analyzed whether the changes in total BCAAs, isoleucine, leucine, and valine levels in serum from baseline to 6 months were associated with the improvement of liver fat content at 12 months (Table 4). We found that reductions in serum total BCAAs, isoleucine, leucine, and valine levels at 6 months were associated with an improvement in liver fat content (Model 3, ß(SE) for total BCAAs: 0.25(0.08), p = 0.001; ß (SE) for isoleucine: 0.98(0.40), p = 0.024; ß(SE) for leucine: 0.80(0.23), *p* < 0.001; ß (SE) for valine: 0.45(0.01), *p* = 0.001) at 12 months after adjustment for age, sex, total energy intake, protein intake, intervention group, HOMA-IR, BMI, liver fat content at baseline, and total BCAAs, isoleucine, and valine levels at baseline and further adjusted for changes in HOMA-IR and BMI from baseline to 6 months. However, there was no significant association between reductions in total BCAAs, isoleucine, leucine, and valine levels and changes in visceral fat and subcutaneous fat content at 6 monthsor 12 months (Table 5).

**Table 5:** Changes(Δ) in adiposity measures at 6 months and 12 months per unit change in BCAAs at 6 months.

	Δtotal BCAAs		ΔIsoleucine		ΔLeucine		ΔValine	
	ß (SE)	*p*	ß (SE)	*p*	ß (SE)	*p*	ß (SE)	*p*
**At 6 months**								
ΔVisceral fat	0.08 (0.30)	0.794	0.24 (1.55)	0.875	0.6 (0.91)	0.472	0.75 (0.56)	0.309
ΔSubcutaneous fat	0.10 (0.34)	0.757	0.56 (1.6)	0.730	0.21 (0.97)	0.827	0.49 (0.59)	0.402
**At 12 months**								
ΔVisceral fat	0.15 (0.30)	0.618	0.77 (1.56)	0.621	− 0.26 (0.92)	0.773	0.54 (0.56)	0.171
ΔSubcutaneous fat	0.01 (0.31)	0.984	− 0.21 (1.61)	0.898	− 0.16 (0.95)	0.871	0.09 (0.58)	0.881

ß (SE) represents changes of the outcomes per change in the circulating metabolite levels during the exercise intervention. Data after adjustment for age, sex, total energy intake, protein intake, intervention group, BMI, value for the respective outcome traits at the baseline examination, total BCAAs, isoleucine, and valine at baseline, ΔHOMA-IR, and ΔBMI. Abbreviations: *HOMA-IR*, homeostatic model assessment of insulin resistance; *BMI* body mass index. ΔHOMA-IR and ΔBMI: changes in HOMA-IR and BMI from baseline to 6 months.

**Figure 1:**
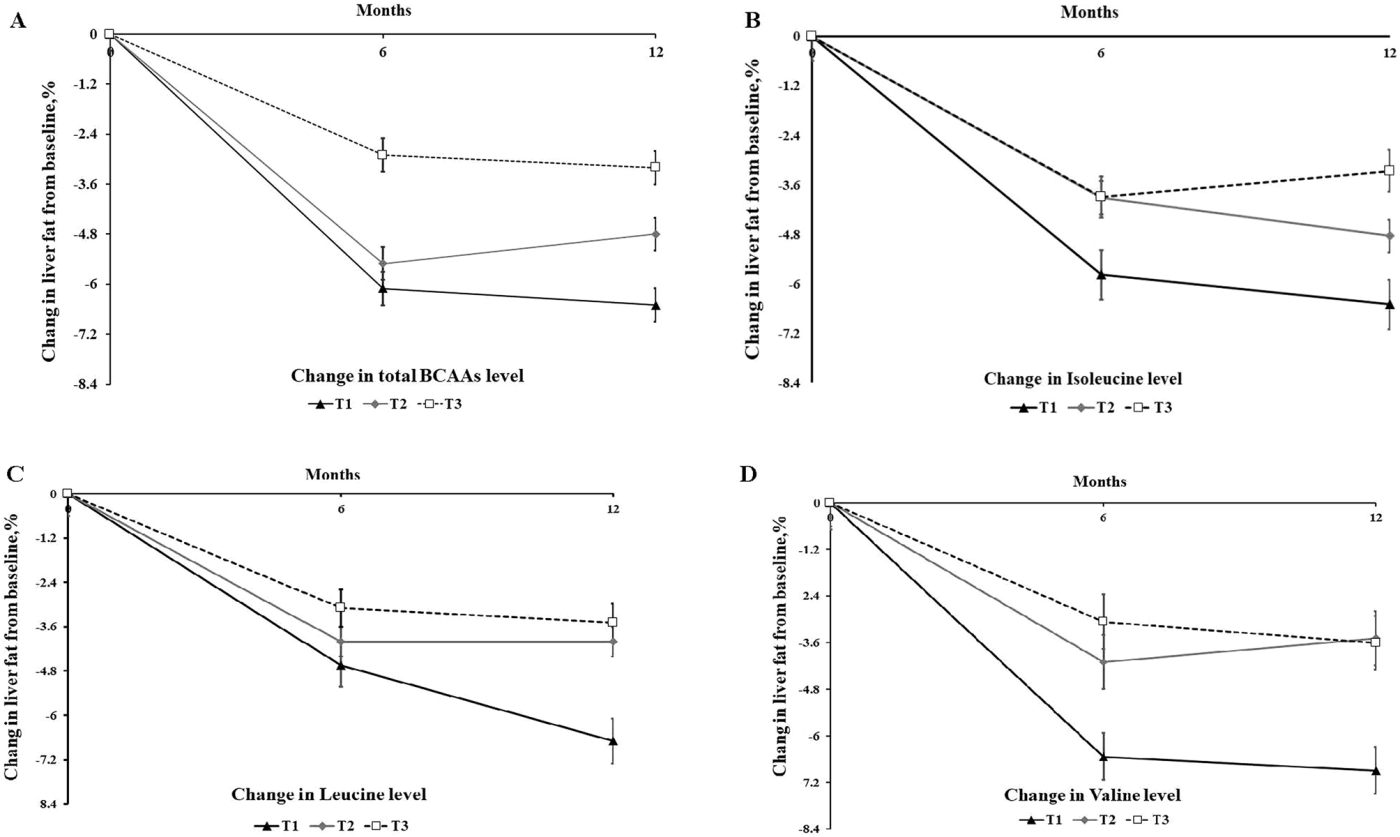
Trajectories of changes in liver fat content according to initial changes in total BCAAs (panels **A**), Isoleucine (panels **B**), Leucine (panels **C**), and Valine (panels **D**). Data were adjusted for age, sex, total energy intake, protein intake, intervention group, HOMA-IR, BMI, liver fat content at the baseline, value for the each metabolites (total BCAAs, Isoleucine, Leucine, Valine) at the baseline, and changes in HOMA-IR and BMI form baseline to 6 months. The lowest tertile (T1) category indicates the largest reduction of circulating metabolites from the baseline to 6 months. Metabolite categories are defined as follow: total BCAAs level-T1, ≤  − 2.8 μg/mL; T2, − 2.8 to 4.7 μg/mL; T3, ≥ 4.7 μg/mL; Isoleucine level-T1, ≤  − 0.55 μg/mL; T2, − 0.55 to 0.88 μg/mL; T3, ≥ 0.88 μg/mL; Leucine level-T1, ≤  − 0.73 μg/mL; T2, − 0.73 to 1.86 μg/mL; T3, ≥ 1.86 μg/mL; Valine level-T1, ≤  − 1.77 μg/mL; T2, − 1.77 to 2.41 μg/mL; T3, ≥ 2.41 μg/mL.

We then investigated the changes in liver fat content during the intervention period by different serum total BCAAs, isoleucine, leucine, and valine levels groups (Fig. 1). We found that individuals with the greatest reduction in total BCAAs level (tertile T1) at 6 months have significantly greater improvement of liver fat content at 12 months after adjustment for confounders compared with another two groups (tertile T2 and tertile T3) (*p* < 0.05). For changes in valine, similar result was found for the improvement of liver fat content at 12 months (*p* < 0.05).

Finally, we investigated whether different exercise intensity could affect the association of changes in total BCAAs, isoleucine, leucine, and valine levels with change in liver fat content. At 6 months, compared to the control group and vigorous-moderate group, concentrations of total BCAAs, isoleucine, and leucine levelsamong moderate exercise group decreased significantly. There was no difference in the change of total BCAAs, isoleucine, orleucine levels between the control group and vigorous-moderate group (Table 6). In multivariable analyses, sex, HOMA-IR, moderate exercise, and changes in BMI were independently associated with changes in total BCAAs (Table 7). We did not find significant interactions between intervention groups (control or moderate exercise or vigorous-moderate) and changes in serum total BCAAs, isoleucine, leucine, and valine levels on change in liver fatcontent (Table 8).

**Table 6:** Effects of moderate and vigorous exercise on plasma concentration of BCAAs (*P* < .008 (0.05/6 comparisons) ) was considered statistically significant.

	Changes (95% CI)			*p* values		
	Control(n = 74)	Moderate exercise(n = 73)	Vigorous-moderateexercise(n = 73)	Moderate Vs Control	vigorous-moderate vs Control	Vigorous-moderate vs Moderate
ΔTotal BCAAs, μg/mL	1.8 (− 2.6 to 6.0)	− 0.7 (− 5.7 to 2.7)	3.0 (0.06 to 6.0)	0.0056	0.5031	0.0008
ΔIsoleucine, μg/mL	0.4 (− 0.6 to 1.1)	− 0.1 (− 0.9 to 0.6)	0.3 (− 0.1 − 1.0)	< .0001	0.1139	0.0006
ΔLeucine, μg/mL	0.7 (− 0.6 to 2.2)	− 0.2 (− 1.6 to 0.9)	1.3 (0.4 to 2.3)	< .0001	0.8250	< .0001
ΔValine, μg/mL	0.8 (− 1.9 to 3.1)	− 0.7 (− 2.9 to 1.4)	1.0 (− 0.2 to 2.9)	< .0001	0.0007	0.0185

**Table 7:** Changes (Δ) in total BCAAs at 6 months.

	Univariate analysis (Δtotal BCAAs)		Multivariate analysis (Δtotal BCAAs)	
	ß(SE)	*p*	ß(SE)	*p*
Age	0.05 (0.07)	0.513		
Sex	0.93 (1.03)	0.364	− 3.60 (1.0)	< 0.001
Total energy intake	− 0.00 (0.00)	0.611		
Protein intake	− 7.34 (15.0)	0.356		
BMI	0.46 (0.16)	0.004		
HOMA-IR	− 0.25 (0.29)	0.397	0.74 (0.29)	0.010
ΔHOMA-IR	0.50 (0.33)	0.133		
ΔBMI	1.09 (0.44)	0.013	1.17 (0.41)	0.004
Moderate exercise	0.77 (1.14)	0.004	2.75 (1.02)	0.007
Vigorous-moderate	− 3.20 (1.14)	0.499	− 1.77 (0.93)	0.055

HOMA-IR, Homeostatic model assessment of insulin resistance; BMI, body mass index. ΔHOMA-IR and ΔBMI: changes in HOMA-IR and BMI from baseline to 6 months.

**Table 8:** Subgroup analyses of associations of change in BCAAs levels with the improvement in liver fat content.

Subgroup	N (%)	ΔLiver fat content at 6 months			ΔLiver fat content at 12 months		
		ß(SE)	*p*	*pinteraction*	ß(SE)	*p*	*p interaction*
Δtotal BCAAs				0.959			0.349
Control	74 (33.6)	0.31 (0.13)	0.081		0.13 (0.14)	0.376	
Moderate exercise	73 (33.2)	0.35 (0.14)	0.014		0.42 (0.15)	0.004	
Vigorous-moderate	73 (33.2)	0.16 (0.10)	0.041		0.12 (0.12)	0.309	
ΔIsoleucine				0.798			0.196
Control	74 (33.6)	0.17 (0.92)	0.784		0.11 (0.68)	0.867	
Moderate exercise	73 (33.2)	1.45 (0.92)	0.117		2.59 (0.94)	0.005	
Vigorous-moderate	73 (33.2)	0.71 (0.46)	0.126		0.38 (0.57)	0.512	
ΔLeucine				0.910			0.324
Control	74 (33.6)	0.47 (0.40)	0.230		0.71 (0.41)	0.081	
Moderate exercise	73 (33.2)	0.97 (0.45)	0.031		1.31 (0.46)	0.005	
Vigorous-moderate	73 (33.2)	0.38 (0.30)	0.198		0.33 (0.37)	0.374	
ΔValine				0.897			0.485
Control	74 (33.6)	0.26 (0.30)	0.123		0.16 (0.29)	0.116	
Moderate exercise	73 (33.2)	0.65 (0.24)	0.007		0.69 (0.25)	0.007	
Vigorous-moderate	73 (33.2)	0.32 (0.17)	0.073		0.27 (0.22)	0.235	

ß (SE) represented changes of the outcomes per change in the circulating metabolite levels during the exercise intervention. Data after adjustment for age, sex, total energy intake, protein intake, HOMA-IR, BMI, liver fat content at the baseline, value for the respective metabolites (total BCAAs, Isoleucine, Leucine,Valine) at the baseline, and changes in HOMA-IR and BMI from baseline to 6 months. Abbreviations: *HOMA-IR* homeostatic model assessment of insulin resistance; *BMI* body mass index.

## Discussion

In this long-term exercise intervention trial, we found that reductions in circulating levels of total BCAAs, isoleucine, leucine, and valine from baseline to 6 months were associated with an improvement of liver fat content at 6 months and 12 months, independent of changes in BMI and HOMA-IR. The associations were not affected by different exercise intensity.

To the best of our knowledge, the present study is the first to investigate the association between changes in BCAAs levels and improvement in liver fat content in response to exercise interventions in NAFLD. Several previous cross-section studies have demonstrated that plasma BCAAs levels are elevated in subjects with NAFLD and that this relationship between BCAAs with NAFLD was independent of several clinical and laboratory covariates including insulin resistance and ß-cell function. Consistently, subjects with more advanced liver damage displayed increased levels of BCAAs^6,17,18^. The levels of BCAAs were associated with the lipid droplets heterogeneity of hepatocytes around zone 3 in patients with NAFLD/NASH^9^. Furthermore, it has been shown that a diet with high BCAAs could induce heterogeneity in the lipid droplets using mouse models of NAFLD and BCAAs may aggravate mitochondrial dysfunction in NAFLD^9,19^, which indicated that impaired BCAAs catabolism may contribute to the occurrence of NAFLD/NASH, and amelioration of BCAAs catabolism could be a new therapeutic approach for NAFLD. In our study, we observed that reductions in circulating levels of total BCAAs, leucine, and valine levels from baseline to 6 months were associated with an improvement of liver fat content at 6 months and 12 months after adjustments for age, sex, total energy intake, protein intake, intervention group, HOMA-IR, BMI, liver fat content, total BCAAs, isoleucine, and valine levels at baseline and further adjustments for changes in HOMA-IR and BMI from baseline to 6 months.

Exercise training is routinely recommended for the treatment and management of NAFLD^20^. Our previous study has shown that vigorous and moderate exercises were equally effective in reducing liver fat^11^. Skeletal muscle is the initial site of BCAAs catabolism^14^. Limited studies have investigated the association between circulating AAs alteration and exercise training. The changes of plasma BCAAs levels during or after exercise have been controversial, which could be unchanged^21,22^, to decrease ^16^, or to increase^23^. The reason for these discrepancies could be explained by different intensity and duration of the exercise. In our study, concentrations of total BCAAs, isoleucine, and leucine levels among moderate exercise group were decreased significantly compared to the control group and vigorous-moderate group. A previous animal study has revealed that branched-chain-amino-acid amino transferase, the first enzyme in the BCAAs catabolism pathway, was activated in the muscles during exercise^15^. Thus, it requires more studies to explore the effects of exercise intensity on BCAAs catabolism and pathogenesis of NASH/NAFLD. In our study, we noticed that the associations of changes in BCAAs levels with changes in liver fat content were not affected by exercise intensity, which indicated reduction in BCAAs levels were associated with improvement in liver fat constantly regardless of different exercise intensity. Furthermore, the present study showed that compared to another two tertiles, individuals with the initial (6-month) greatest changes in total BCAAs levels (tertile 1) gained significantly better improvement of liver fat content at 12 months, which suggested that change in BCAAs levelsmay function as a marker to predict improvement of liver fat content. A longitudinal study found that baseline valine levels are predictive of longitudinal changes in liver fat accumulation in obese children. ^7^.

The major strength of this study included analyses of dynamic changes with repeated measurement of circulating isoleucine, leucine, and valine levels from baseline to 6 months. Assessment of liver steatosis was performed by a validated, quantitative, non-invasive MRI method, and repeated to measure longitudinal changes in hepatic fat accumulation. The hepatic fat content quantified by magnetic resonance spectroscopy washighly reproducible and correlated with the histologic features of fibrosis and steatosis^24,25^.

Several limitations existed in the present study. First of all, dietary calorie and fat intake were not controlled in this study becausewe aimed to examine the isolated effects of exercise on NAFLD. During the 12-month intervention, dietary intake of energy and macronutrients was not significantly different among different intervention groups. Secondly, participants in the study were overweight or obese adults Chinese. Further research would be necessary to confirm our findings in a general population. Finally, we could not determine whether plasma BCAAs were the causal factor of changes in hepatic fat content or just the biomarkers of metabolic dysfunction.

In conclusion, we found that reductions in circulating BCAAs levels were significantly associated with an improvement of liver fat content after exercise intervention, independent of BMI and HOMA-IR changes. The associations were not modified by different exercise intensity. Further studies invarious populations are necessaryto validate our findings.

## Materials and methods

### Study subjects

The effect of Moderate and Vigorous Exercise on Nonalcoholic Fatty Liver Disease wasa 1-year randomized intervention trial. The study design and sample collection have previously been described^11^. The eligibility criteria were participants aged 40–65 years with central obesity (waist circumference ≥ 90 cm in men and ≥ 85 cm in women) and NAFLD which was confirmed their diagnosis by proton magnetic resonance spectroscopy (IHTG content ≥ 5%). Participants were excluded if they consumed more than a mean of 140 g of ethanol (10 alcoholic drinks) per week in men and 70 g of ethanol (5 drinks) in women during the past 6 months. Patients were also excluded if they had a history of acute or chronic viral hepatitis, drug-induced liver diseases, or autoimmune hepatitis. In addition, patients were excluded if they had a history of diabetes, uncontrolled hypertension, chronic kidney disease, hyperthyroidism, myocardial infarction within 6 months, or heart failure (New York Heart Association class III or IV). Furthermore, patients were excluded if they were participating in weight loss programs orhad a medical condition that limited their exercise capability. In brief, a total of 220 individuals with central obesity and NAFLD were selected and randomly assigned to control group (n = 74, be instructed to not change their physical activity routine), moderate exercise group (n = 73, brisk walking 150 min per week at 45–55% of maximum heart rate for 12 months), and vigorous-moderate exercise group (n = 73, jogging 150 min per week at 65–80% of maximum heart rate for 6 months and brisk walking 150 min per week at 45–55% of maximum heart rate for another 6 months). Of the total 220 individuals, the current study included 208 participants on the basis of availability of blood samples and measurements of BCAAs levels at the baseline examination and at 6 months during the intervention. All study participants were instructed to not change their diet and not intake BCAA supplementation.

### Measurements of liver fat and anthropometrics

The liver fat content was measured using proton magnetic resonance spectroscopy (MRI)^26^. Abdominal visceral fat and subcutaneous fat areas were measured by computed to mography (CT). Height and Body weight was measured in the morning before breakfast. Body mass index (BMI) was calculated as body weight in kilograms divided by the square of height in meters (kg/m^2^). All study outcomes were measured at baseline and 6- and 12-follow-up visits.

### Measurements of serum BCAAs

Blood samples were collected in the early morning after an overnight fasting; serum samples were stored at − 80 °C until analysis. Blood concentrations of BCAAs (leucine, isoleucine, and valine) were measured following this procedure: 1 µL L-phenyl-d5-alanine (10 µg/mL) and 80 µL acetonitrile were added into 20 µL of serum. The mixture was vortex-mixed for 30 s and centrifuged at 19,000 g$\times$ for 10 min at 4 °C. 40 µL of the supernatant was then mixed with 120 µL water, the aliquot of which (5 µL) was injected into the LC–MS/MS system for analysis. Data of BCAAs (leucine, isoleucine, and valine) concentration were acquired and processed using Agilent Mass Hunter Workstation Data Acquisition and Quantitative Analysis B.07.00 (Agilent Technologies, Santa Clara, CA, USA).

### Other measurements

Nutrient intake was estimated by 3 consecutive 24-h dietary recalls (2weekdays and 1weekend day) at baseline, 6 and 12 months, respectively. Nutrient intake was calculated based on the nutrient content listed in the Chinese Food Composition Table. Physical activity was assessed using the International Physical Activity Questionnaire (long form) at the baseline examination^27^. Homeostatic model assessment of insulin resistance (HOMA-IR) was calculated as follows: Fasting plasma insulin (mU/L) *fasting plasma glucose (mmol/L)/22.5.

### Statistical analyses

Data were summarized using frequencies and counts for categorical variables and means and standard deviations (SD) for continuous variables. We first analyzed associations of total BCAAs, leucine, isoleucine, or valine levels with liver fat content at the baseline using generallinear model adjusted for age, sex,total energy intake, protein intake, intervention group, HOMA-IR, and BMI at baseline. Secondly, we calculated changes in total BCAAs, leucine, isoleucine, and valine levels from baseline to 6 months during the intervention and used general linear models to analyze associations between changes in these metabolites and changes in liver fat contentat 6 months and 12 months. Multivariate-adjusted models were performed: Model 1 adjusted for age, sex, total energy intake, protein intake, intervention groups, HOMA-IR, BMI, liver fat content at baseline, total BCAAs, isoleucine, and valine levels at baseline respectively; Model 2 was further adjusted for change in HOMA-IR from baseline to 6 months; and Model 3 was additionally adjusted for change in BMI from baseline to 6 months. To determine whether associations of changes in metabolites with the improvement in liver fat content can be modified by different exercise intensity, we added an interaction term of intervention groups (control or moderate exercise or vigorous exercise) and change in metabolites in generalized linear models. Statistical analyses were performed with SAS 9.4 software (SAS Institute). All *p* values were nominal and two-sided, and a *p* value, 0.05 was considered statistically significant.

### Ethics statement

This study was approved by the ethics committee of the First Affiliated Hospital of Xiamen University and conducted in accordance with the rules of the Declaration of Helsinki of 1975, revised in 2013.All participants gave written informed consent.

## Data Availability

The original contributions presented in the study are included in the article, further inquiries can be directed to the corresponding author.
